# Transcriptomic Association Analysis of the Metabolic Mechanism of Sulfamethoxazole in Channel Catfish (*Ictalurus punctatus*)

**DOI:** 10.3390/ani14071059

**Published:** 2024-03-30

**Authors:** Xiangxuan Du, Ruyu Sun, Lei Zhang, Yongtao Liu, Xiaohui Ai

**Affiliations:** 1 College of Fisheries and Life Science, Shanghai Ocean University, Shanghai 201306, China; assisic@163.com (X.D.); sunruyu_1@163.com (R.S.); zl521199@163.com (L.Z.); aixh@yfi.ac.cn (X.A.); 2 Yangtze River Fisheries Research Institute, Chinese Academy of Fishery Sciences, Wuhan 430223, China; 3 Hubei Province Engineering and Technology Research Center for Aquatic Product Quality and Safety, Wuhan 430223, China; 4 Key Laboratory of Control of Quality and Safety for Aquatic Products, Ministry of Agriculture, Beijing 100141, China

**Keywords:** channel catfish, liver, sulfamethoxazole, transcriptome

## Abstract

**Simple Summary:**

Sulfamethoxazole is a commonly used antimicrobial drug in aquaculture. In order to understand its effect on the liver transcriptome of channel catfish, we orally administered a certain proportion of sulfamethoxazole into the fish and analyzed the changes in the transcriptome to determine the effect of the drug on the fish. By studying changes in the transcriptome, we found that the drug acted on drug metabolizing functions in the liver. Stimulated fish try to ensure the normal expression of their physiological functions by accelerating the operation of various metabolisms. The detoxification process is facilitated by promoting glucuronidation in the fish liver; while lipid metabolism is increased, the antioxidant pathway is affected, and the glucose xenobiotic pathway is enhanced in order to maintain energy homeostasis and to ensure a normal energy supply for metabolism. Other cellular metabolisms are also carried out to repair damaged normal organisms. This study provides a reference for understanding drug metabolism in channel catfish and provides a warning about the use of the drug.

**Abstract:**

Sulfamethoxazole is a widely used antimicrobial drug used to treat bacterial diseases in aquaculture. To understand the gene expression in channel catfish liver after treatment with sulfamethoxazole, in this study, the treatment group received sulfamethoxazole (100 mg/kg bw), which was administered orally once, and samples were taken at 5 h, 12 h, and 6 d after the administration of sulfamethoxazole, while the control group was orally administered sterile water. To further identify potentially significant genes, a transcriptome analysis using RNA-seq was carried out. More than 50 million high-quality reads were found. After filtering and quality analysis, these reads were identified as 54,169,682, 51,313,865, 51,608,845, and 49,333,491. After counting 23,707 of these transcripts for gene expression, it was discovered that 14,732 of them had genes with differential expression. Moreover, we found that the annotation with the most GO variation was “cellular process” (1616 genes), “metabolic process” (1268 genes), “binding” (1889 genes), and “catalytic activity” (1129 genes). KEGG pathways showed that the “metabolic pathway” was the pathway that was significantly enriched in both experimental groups when comparing the experimental groups: 5 h and 12 h (128 genes); 5 h and 6 d (332 genes); and 12 h and 6 d (348 genes). Also, UDP- glucuronosyltransferase (*ugt*), which is associated with glucuronidation, and UDP-glucuronosyltransferase 2C1-like (*ugt2a1*) showed significant upregulation. Carboxylesterase 5A-like (*ces3*), which promotes fatty acyl and cholesteryl ester metabolism, and the glutathione transferase family were upregulated in the expression of sulfamethoxazole metabolism in the liver, which significantly affected the metabolic effects of the drug. Meanwhile, *dypd, uck2b*, and *rrm2,* which are related to nucleotide synthesis and metabolism, were upregulated. Our study extends the knowledge of gene expression in drug metabolism in channel catfish and further provides insight into the molecular mechanism of sulfamethoxazole metabolism.

## 1. Introduction

Channel catfish (*Ictalurus punctatus)*, a sizeable warm-water fish with a long and broad body, is an economically significant fish in China due to its diet, fast growth, wide adaptability, and excellent meat quality [1]. The China Fisheries Statistics Yearbook 2023 reports that the output of channel catfish was 363,344 and 416,200 tons in 2021 and 2022, respectively. Still, as the scale and density of farming have increased, several diseases, particularly those brought on by viruses [2], bacteria [3], and parasites [4], as well as their combined effects [5], have emerged. These diseases lead to great mortality rates, causing significant economic losses. There are no specific drugs to treat sick fish; workers treat sick fish with a multitude of antimicrobial medications frequently, leading to hazardous drug-related issues [6]. Antimicrobial agent use has become important as an industry component for addressing these issues. Drug abuse is linked to potential safety issues, and there is a necessity for a new generation of medications as pathogens develop drug resistance [7]. The most crucial task is to research sulfamethoxazole metabolism and choose the best medications for the prevention and treatment of channel catfish sickness to prevent drug misuse and ecological damage.

Currently, sulfamethoxazole is used as the broad-spectrum antibiotic medication to treat bacterial illnesses in aquaculture. Sulfamethoxazole has a bactericidal effect because it is structurally similar to p-aminobenzoic acid (PABA), which can act competitively with PABA on dihydrofolate synthase in bacteria, preventing the synthesis of dihydrofolate in bacteria and thus inhibiting the growth and reproduction of bacteria. Additionally, it halts the production of bacterial DNA, thymidine, and purines [8]. The general pharmacokinetics of sulfamethoxazole have been studied. Sulfamethoxazole reached high residual levels in the liver following oral dosing. Sulfamethoxazole showed quick absorption and widespread dispersion according to pharmacokinetics research [9]. In recent years, studies have shown that sulfamethoxazole has a complex structure [10], a low biodegradation rate, and a long residual time after use; further, its long-term accumulation in aquatic environments can have specific effects on humans. It also has an inhibitory effect on the growth of fish; sulfamethoxazole can affect the development of fish by acting on the activity of metabolic enzethoxy resorufinorufin O-deethylase, (EROD) and antioxidant enzymes (superoxide dismutase, SOD, and catalase (CAT)) in fish [11]. Many studies have investigated sulfamethoxazole in aquatic products [12], but the metabolic effects of sulfamethoxazole in channel catfish still need to be discovered. Here, we used high-throughput RNA sequencing (RNA-seq) technology to perform an analysis of transcriptomic metabolic genes.

The liver is a vital organ for preserving the body’s regular metabolic processes since it has immunological, coagulation, and metabolic activities. The liver is the main organ where drugs are metabolized. It has a more prolonged exposure duration and higher drug concentration than other organs [13] and can metabolize medicines through various metabolic routes. Innate immunity relies heavily on the liver, and the liver’s cDNA library is more varied than the one created in blood cells. RNA-seq is an indispensable tool for analyzing differential gene expression (DEG) at the whole transcriptome level and for studying mRNA differential splicing, which can help to understand gene function at various levels while obtaining DEG matrices for various analyses. RNA-seq is now widely used in the process of analyzing the transcribed genomes of aquatic organisms [13] due to its benefits of more accurate quantification, higher reproducibility, a broader detection range, more reliable analysis, the discovery of new transcripts, and allele-specific gene expression. As a result, we can trust the results of our analysis of the gene alterations in this experiment. In this study, we developed a high-throughput RNA sequencing approach through the liver to examine alterations in the catfish’s liver transcriptome following oral sulfamethoxazole dosing. This method offers the groundwork for the sensible use of medications in future aquaculture species to avoid the potential harm caused by the abuse of a particular drug. It is more accurate and representative of the analysis of the impacts of drug metabolism.

The aim of this study was to elucidate the effect of the transcriptome on drug metabolism in the organism by observing the changes of the drug on the hepatic transcriptome after oral instillation of sulfamethoxazole, to reveal the mechanism of action of sulfamethoxazole and to provide a new study on the action of the drug on the changes in the hepatic genome.

## 2. Materials and Methods

### 2.1. Maintenance and Treatment of Channel Catfish

A total of 50 healthy and moderate size (180 to 200 g) channel catfish were provided from the Yangtze River Fisheries Research Institute of the Chinese Academy of Fishery Sciences (Wuhan, China). The fish were randomly divided into two groups, the treatment group (T) and the control group (CK). Fish in the T were orally treated with sulfamethoxazole at a single dose of 100 mg per kg body weight (100 mg/kg bw) and fish in the CK were gavaged with sterile water. The doses were orally administrated by inserting a preloaded syringe down the esophagus [14]. Samples were taken at 5 h (peak concentration, T_1_), 12 h (half of the peak concentration, T_2_), and 6 d (the time point when the concentration fell back to the baseline, T_3_) after treatment. Three viable and healthy individuals were selected at each time point from T and CK, respectively. The fish were first anesthetized with tricaine methanesulfonate (MS-222, 200 mg/L) [15]. Afterwards, the fish was placed on ice and its abdomen was cut open using scissors; then, the skin and muscles were carefully incised with a scalpel to expose the internal organs, immediately followed by the gentle removal of the liver tissue using forceps. Then, they were put into labeled freezing tubes aseptically, and a total of 12 samples were rapidly frozen with liquid nitrogen and stored in the ultra-freeze at −80 °C for future sample preparation and analysis.

### 2.2. RNA Isolation, RNA-seq Library Construction and Sequencing

Total RNA was extracted using TRIzol reagent (Invitrogen, Carlsbad, CA, USA) in accordance with the manufacturer’s protocol. In addition, in order to ensure the quality of sequencing, we use strict quality control measures to control the quality of library construction, and the test standards are as follows: Rnase-free agarose gel electrophoresis was used to analyze the integrity of RNA and the presence of DNA contamination; a Nanophotometer spectrophotometer was used to detect the purity of RNA (OD260/280 and OD260/230 ratio); a Qubit2.0 Fluorometer was used for the accurate quantification of RNA concentration; and an Agilent 2100 bioanalyzer (Agilent Technologies, Palo Alto, CA, USA) was used for the accurate detection of RNA integrity, which automatically measures the 28 s:18 s rRNA ratio and computes an RNA integrity number (RIN). A total of 12 samples were used for library construction and Illumina RNA-seq. RNA samples with high purity and high integrity were selected and used for cDNA library construction. After extraction of total RNA, we performed the library construction. Eukaryotic mRNAs with polyA tails were enriched by magnetic beads with Oligo(dT) and then the mRNAs were broken up with buffer. The first strand of cDNA was synthesized in the M-MuLV reverse transcriptase system using fragmented mRNA as a template and random oligonucleotides as primers. Subsequently, the RNA strand was degraded with RNaseH and the second strand of cDNA was synthesized with dNTPs in the DNA polymerase I system. The purified double-stranded cDNA was end-repaired, A-tailed, and ligated to the sequencing junction, and the cDNA of about 200 bp was screened using AMPure XP beads and amplified using PCR. The PCR product was purified using AMPure XP beads again, and the library was finally obtained. After library construction, sequencing was conducted using Illumina Novaseq6000 by Gene Denovo Biotechnology Co. (Guangzhou, China). In order to ensure the data quality, we performed quality control on the downstream raw reads using fastp [16], which is designed to detect contamination by aligning the reads against a collection of reference genomes using a small percent (2% by default) of reads for fast screening. To remove the reads containing adapter, we used an N ratio greater than 10% with all A bases and filtered the low quality data to obtain clean reads. Meanwhile, Q20, Q30, and GC contents of the clean data were calculated.

### 2.3. Differential Expression of Genes Analysis (DEGs)

We used the short reads alignment tool bowtie2 [2] to align the clean reads to the channel catfish ribosome database (Silva Reference Database: GQ465834), divided the reads from the aligned ribosomes, and used the retained unmapped reads for subsequent transcriptome analysis. The clean data were mapped utilizing the assembled greater channel catfish genome (NCBI Reference Sequence: NC_003489.1) as a reference. Hisat2 was used to construct an index of the reference genome, and bipartite clean reads were compared to the reference genome. To obtain the expression level of each transcript, the number of reads was counted using FeatureCounts (featureCounts (subread) v2.0.1) [16] to map each gene. The fragments per kilobase of exon model per million mapped fragments (FPKM) method was used to calculate the expression of a single gene, which eliminates the effect of different gene lengths and sequence levels on the calculation of gene expression [17]. Differential expression analysis of RNA between groups was performed using DESeq2 (1.42.0) [18] software, and edgeR (R4.2.2) [19] software was used between samples. The analysis was divided into three parts: standardization of the read count (normalization); calculation of the hypothesis test probability (*p*-value) according to the model; and finally, correction of the multiple hypothesis test, which was performed to obtain the FDR value (false discovery rate). The results with *p*-value < 0.05 and |log2 (Fold Change)| > 1 were identified as differentially expressed genes (DEGs).

### 2.4. GO Functional Enrichment Analysis for DEGs

We annotated the DEGs to analyze the potential consequential changes of function following sulfamethoxazole treatment. Firstly, all DEGs were mapped to GO terms in the Gene Ontology database (http://www.geneontology.org/). Gene numbers were calculated for every term, and significantly enriched GO terms in DEGs compared to the genome background were defined using a hypergeometric test. The formula for the calculation of the *p*-value is as follows:

(1) $$P=1-\sum_{i=0}^{m-1}(\frac{\left(\begin{array}{c} M\\ i \end{array}\right)\left(\begin{array}{c} N-M\\ n-i \end{array}\right)}{\left(\begin{array}{c} N\\ n \end{array}\right)})$$

Here, N is the number of all genes with GO annotation; n is the number of DEGs in N; M is the number of all genes annotated to specific GO terms; and m is the number of DEGs in M. The calculated *p*-value (*p* ≤ 0.05) went through FDR correction, taking FDR ≤ 0.05 as a threshold. GO terms that satisfy this condition are defined as those that are significantly enriched in differentially expressed proteins and GO functional significance enrichment analysis identifies the main biological functions performed by differentially expressed proteins.

### 2.5. Pathway Analysis of DEGs Enrichment

Genes usually interact with each other to play roles in certain biological functions. Pathway-based analysis helps to understand genes’ biological functions further. KEGG [20] is the major public pathway-related database. When compared to the whole genome background, DEGs’ metabolic or signal transduction pathways were found to be highly enriched using pathway enrichment analysis. The calculation formula is identical to the one used in the GO analysis. Here, N is the number of all genes with KEGG annotation, n is the number of DEGs in N, M is the number of all genes annotated to specific pathways, and m is the number of DEGs in M. The calculated *p*-value (*p* ≤ 0.05) went through FDR correction, taking FDR ≤ 0.05 as a threshold. Pathways meeting this condition were defined as significantly enriched pathways in DEGs.

### 2.6. Quantitative Real-Time Reverse

Transcription PCR (qRT-PCR) was used to confirm the expression levels of DEGs identified from the RNA-seq analysis. The qRT-PCR primers were created using Primer Premier 5.0, and the PCR reaction technique and fluorescence quantification reaction system were carried out with Livak [21]. Using the TRIzol Reagent Kit, total RNA was extracted from each group of liver samples, and cDNA was produced using reverse transcription in accordance with the directions included with the RT SuperMix for qPCR (+gDNA wiper) kit (Vazyme, Guangzhou, China). The 10 μL qRT-PCR system consisted of 5 μL of ChamQ SYBR qPCR premix (GDSBIO, Guangzhou, China), 0.2 μL of each primer (10 μM), 1 μL of template cDNA, and 3.6 μL of ddH2O. LightCyeler 480 Multiwell Plate 384 (Roche, Ludwigshafen, Germany) was used for qRT-PCR. qRT-PCR was performed with the following reaction program: pre-denaturation at 95 °C for 30 s, followed by 40 cycles of 95 °C for 3 s, and 60 °C for 30 s. The reaction was carried out with the LightCyeler 480 Multiwell Plate 384 (Roche Ludwigshafen, Germany). Each sample had three replicates, and the data were processed using the 2^−ΔΔct^ method to create histograms of relative gene expression. The primers used are listed in Table 1.

## 3. Results

### 3.1. Illumina Sequencing and Quality Assembly

To investigate the effect of sulfamethoxazole administration on the transcriptome of the liver of channel catfish, we performed RNA-seq using the Illumina sequencing platform. In the transcriptome sequencing process, the control and experimental groups produced 163,287,864 and 458,842,604 raw reads, respectively. Following filtering tests on the raw reads, the control and treatment groups yielded 162,509,046 and 456,768,604 clean reads, respectively. The trimmed reads with the trimmed rate were 99.08% and 99.09%, respectively. And the number of bases with sequenced base quality values reaching the level of Q20 or higher in the filtered data was above 97.82% of clean data, and Q30 was above 93.84%. The mean values of GC percentages were 47.48% and 47.04%, respectively (Table 2), indicating successful sequencing of the channel catfish liver transcriptome. Trimmed reads were then used for the subsequent analysis.

### 3.2. Comparative Analysis with the Reference Genome

The overall numbers of reads in the control and experimental groups were 162,365,712 and 456,423,586, respectively. The total number of reads that could be compared to the reference sequences were 149,519,355 and 420,455,093, accounting for more than 91% of the total reads. Additionally, 5.01%, 4.57%, 4.51%, and 4.84% of reads could be matched to different places in the reference sequence. It shows that sulfamethoxazole therapy significantly impacts the channel catfish transcriptome of the liver genes, providing rich data for further screening and analysis of DEGs (Table 3).

### 3.3. Analysis of DEGs

**Figure 1:**
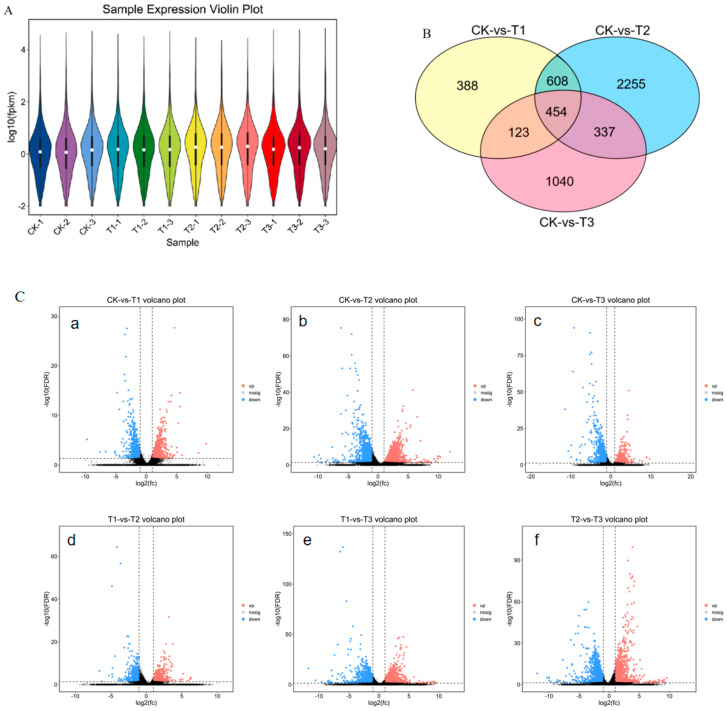
Sample relationship analysis of RNA-seq. (**A**) The violin plot of each sample. The white dot represents the median; the black rectangle is the range from the lower quartile to the upper quartile; the black line through the violin chart represents the confidence interval; the outer shape of the black rectangle is the kernel density estimation; the length of the vertical axis of the graph represents the degree of data dispersion; and the length of the horizontal axis represents how much data are distributed at a certain vertical coordinate position. (**B**) The Venn diagram of DEGs in CK-vs.-T1, CK-vs.-T2, and CK-vs.-T3. (**C**) The volcano plot of DEGs in CK-vs.-T1(**a**), CK-vs.-T2 (**b**), CK-vs.-T3 (**c**), T1-vs.-T2 (**d**), T1-vs.-T3 (**e**), and T2-vs.-T3 (**f**). Red dots represent upregulated genes (up), blue dots represent downregulated genes (down), and blue dots represent genes that do not meet the threshold screening (nodiff); The horizontal axis is Log2 (fold change), where the more off-centre the point is, the greater the magnitude of the difference; The vertical axis is Log 10 (*p*-value), where the more points are towards the top of the plot, the more significant the difference.

The transcript abundance of genes was estimated using fragments per kilobase of exon model per million mapped fragments (FPKM), and based on the FPKM values of each gene, we show the expression distribution of different sample genes or transcripts using expression distribution maps. To study the DEGs of each group for comparison, DESeq2 (1.42.0) [18] software was used to analyze the genes with FDR < 0.05 and |log2FC| > 1 as significantly different genes to generate gene expression profiles. Figure 1A shows that the median and quartile values of the gene transcript levels across the libraries compared for different expressions were comparable. Through pairwise comparisons, a total of 1573, 3654, and 1954 DEGs were identified between CK and T1, CK and T2, and CK and T3, respectively (Figure 1B). Among these DEGs, 955 genes were upregulated, and 618 genes were downregulated in the control and 5 h experimental groups (CK-vs.-T1); 2428 genes were upregulated and 1226 genes were downregulated in the control and 12 h experimental groups (CK-vs.-T2); 993 genes were upregulated and 961 genes were downregulated in the control and 6 d experimental groups (CK-vs.-T3). There were 1344 DEGs detected in the 5 h and 12 h (T1-vs.-T2) experimental groups, of which 935 genes were upregulated and 409 genes were downregulated. At 5 h and 6 d (T1-vs.-T3), 2824 DEGs were found in the experimental group, 1632 genes had their expression upregulated, and 1192 genes had it downregulated. A total of 3383 DEGs were detected in the experimental group at 12 h and 6 d (T2-vs.-T3), 1401 genes were significantly upregulated, and 1982 genes were downregulated (Figure 1C).

### 3.4. Gene Ontology (GO) Annotation of Differentially Expressed Genes (DEGs)

**Figure 2:**
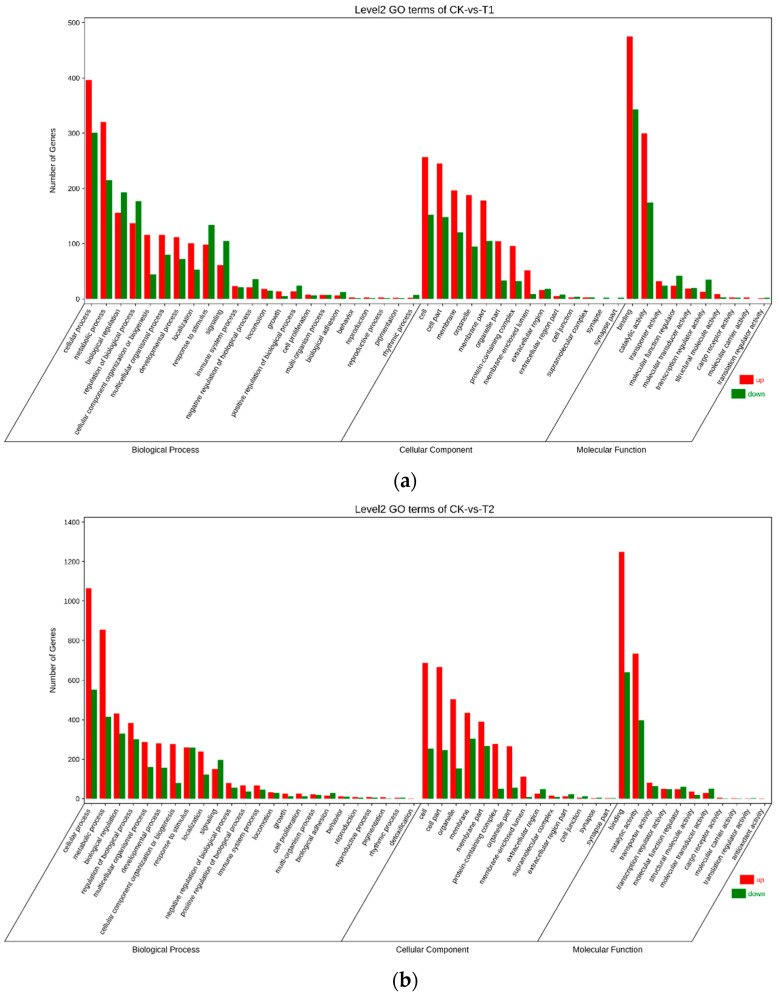

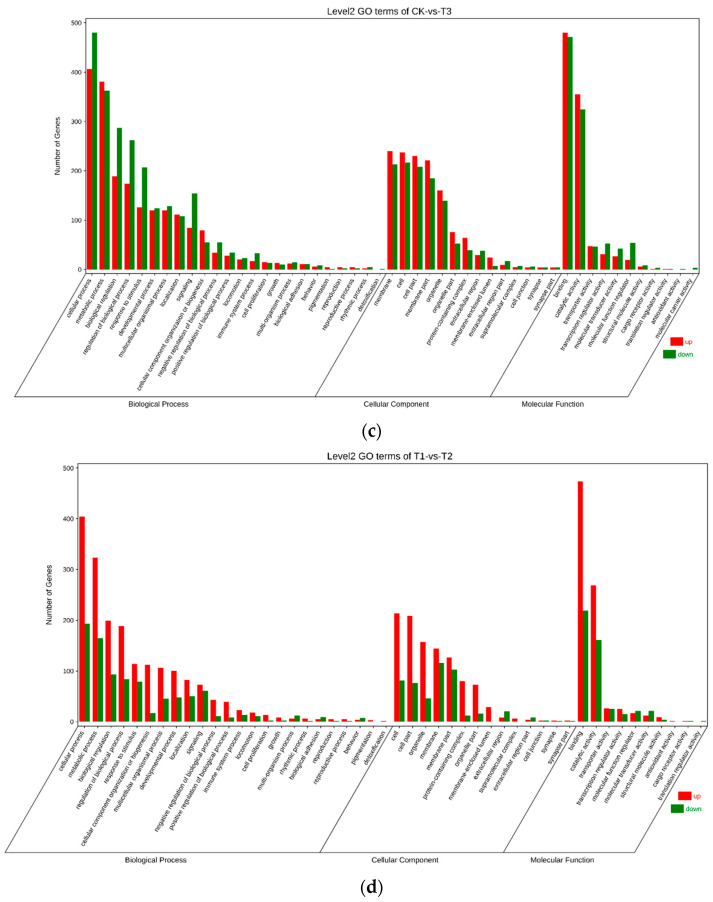

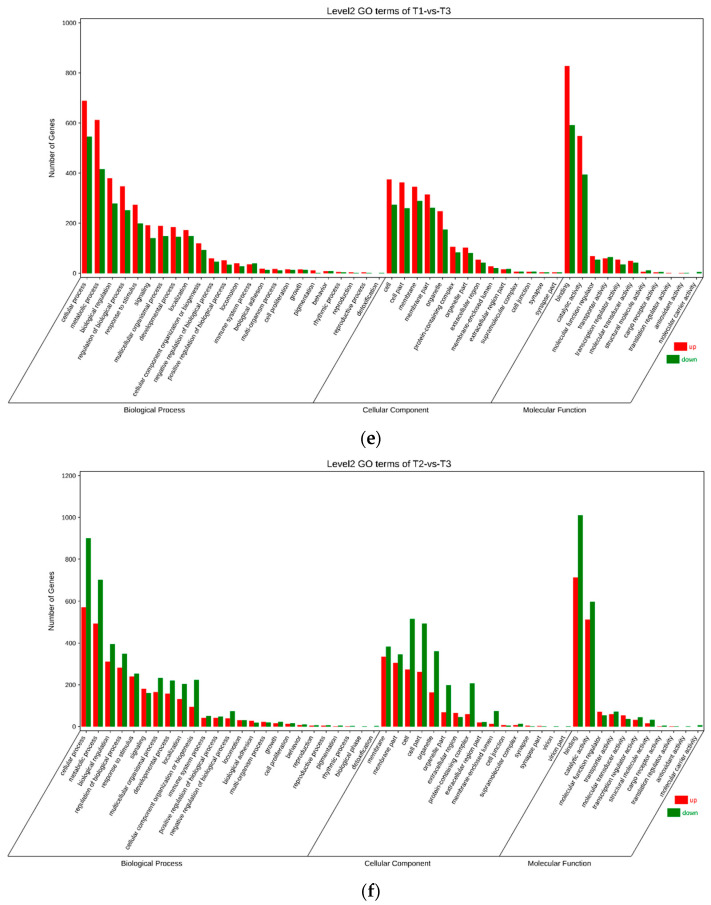
Histogram of the effect of sulfamethoxazole on liver GO levels in channel catfish. (**a**): control and 5 h experimental group (CK-vs.-T1); (**b**): control and 12 h experimental group (CK-vs.-T2); (**c**): control and 6 d experimental group (CK-vs.-T3); (**d**): 5 h versus 12 h (T1-vs.-T2); (**e**): 5 h versus 6 d (T1-vs.-T3); and (**f**): 12 h versus 6 d (T2-vs.-T3). The X-axis is the enriched GO term; the Y-axis is the vertical coordinate, which is the number of differential genes in the term. Different colors are used to distinguish between biological processes, cellular components, and molecular functions.

An international standard for gene functional classification, Gene Ontology (GO) [22], provides a rigorously defined concept and a dynamically updated regulated vocabulary to fully characterize the characteristics of genes and their products in every organism. To determine the functional definition of the samples, the EDGs were projected into the GO database and categorized into molecular function (MF), biological process (BP), and cellular component (CC) based on their respective roles. Each figure displays secondary classifications of the top 20 GO keywords with the most annotations (Figure 2). A GO enrichment analysis was performed on the DEGs in the channel catfish, and significant enrichment was determined by a corrected *p*-value < 0.05. Significant alterations in the biological process category were seen in the “cellular process” (697 genes) and “metabolic process” (535 genes) in the results of the control group compared with the 5 h (CK-vs.-T1) experimental group. “cell” (409 genes) and “cell part” (393 genes) were discovered in the cellular component category. The molecular function category includes “binding” (818 genes) and “catalytic activity” (474 genes). The enrichment of genes in the control group compared to the 12 h (CK-vs.-T2) group was similar to that of the 5 h (CK-vs.-T1), with significant aggregation in the biological processes of “cellular process” (1616 genes), and “metabolic process” (1268 genes). In the cellular component category, it is “cell” (941 genes) and “cell part” (911 genes). In the molecular function category, it is “binding” (1889 genes) and “catalytic activity” (1129 genes). The most altered terms in the biological process category in the comparison between the control group and 6 d (CK-vs.-T3) were “cellular process” (886 genes), “metabolic process” (743 genes), and “cell” (454 genes), “membrane” (453 genes) in the cellular component category, and “binding” (951 genes) and “catalytic activity” in the molecular function category (679 genes). The expression was concentrated in the catalytic phase of substance metabolism, in the part of cellular functional processes and binding associated with membrane organelles. Each experimental group was compared to the control group. This suggests that the drug’s effect on the liver following the experimental treatment is related to metabolic cycling and substance delivery. The terms “cellular process” (597 genes) and “metabolic process” (488 genes) were most frequently allocated to the biological process category in the 5 h experimental group compared to the 12 h experimental group (T1-vs.-T2). It is the “cell” (295 genes) and “membrane” (285 genes) in the cellular component category. It is “binding” (693 genes) and “catalytic activity” (430 genes) in the molecular function category. In the comparison between the 5 h experimental group and the 6 d experimental group (T1-vs.-T3), “cellular process” (1234 genes) and “metabolic process” (1028 genes) in the biological process category, “cell” (648 genes) and “membrane” (634 genes) in the cellular component category, “binding” (1419 genes) and “catalytic activity” (943 genes) in the molecular function category were the most assigned components. Genes were primarily concentrated in the biological process category in the “cellular process” (1471 genes) and “metabolic process” (1195 genes), “cell” (788 genes) and “cell part” (755 genes) in the cellular component category, and “binding” (1724 genes) and “catalytic activity” (1110 genes) in the molecular function category in the comparison between the 12 h and 6 d experimental groups (T2-vs.-T3). In all experimental groups, the medication’s impact on cellular membrane reaction pathways was consistently evident, impacting the circulation related to drug metabolism.

### 3.5. KEGG Pathway Analysis of DEGs

**Figure 3:**
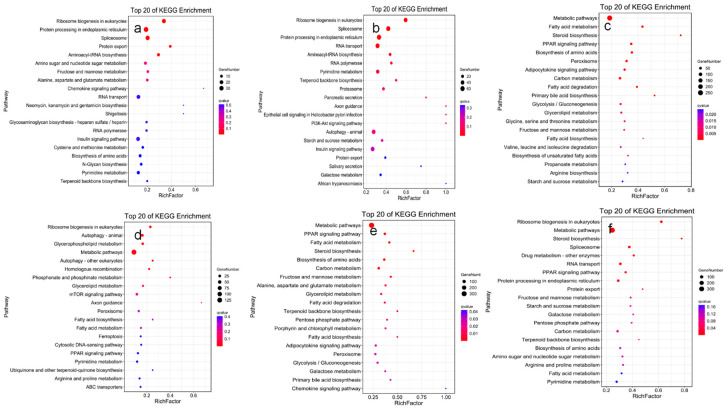
Histogram of the effect of sulfamethoxazole on liver KEGG levels in channel catfish. (**a**): control and 5 h experimental group (CK-vs.-T1); (**b**): control and 12 h experimental group (CK-vs.-T2); (**c**): control and 6 d experimental group (CK-vs.-T3); (**d**): 5 h versus 12 h experimental group (T1-vs.-T2); (**e**): 5 h versus 6 d experimental group (T1-vs.-T3); and (**f**): 12 h versus 6 d experimental group (T2-vs.-T3). X-axis, pathway name; Y-axis, Rich factor. The size of the dot indicates the number of DEGs in this pathway, and the dot’s color corresponds to the different q value ranges.

We analyzed the active biological pathways in our samples to investigate biological behavior. DEGs were mapped to the KEGG database and linked to essential pathways such as metabolism and signal transduction based on the entire transcriptome background [23]. These DEGs were significantly enriched by the *q* value ≤ 0.05 statistical test (Figure 3). In the control group compared with the 5 h experimental group (CK-vs.-T1), the DEGs were distributed in 176 signaling pathways, and the most annotated DEG pathway was “Protein processing in endoplasmic reticulum” (36 genes). The DEGs in the control group and 12 h (CK-vs.-T2) were spread throughout 242 signaling pathways, with “Protein processing in endoplasmic reticulum” (62 genes) having the highest annotation count, similar to the 5 h experimental group. The DEGs in the control group vs. 6 d (CK-T3), 5 h vs. 12 h (T1-T2), 5 h vs. 6 d (T1-T3), and 12 h vs. 6 d (T2-T3) were distributed in 185, 170, 220, and 216 signaling pathways, respectively, of which the most annotated pathways were all “Metabolism pathways”. Thus, the functional enrichment analysis of DEGs proposes that sulfamethoxazole predominantly affects the hepatic metabolic cycle, and further testing will focus on these pathways.

### 3.6. Verification of the Differential Expression of DEGs

**Figure 4:**
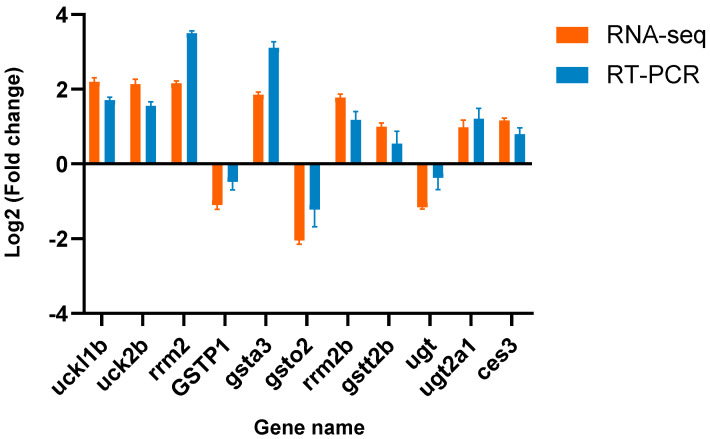
Comparing the results of RNA-seq with RT-PCR for gene expression levels. Genes up- or downregulated after treatment with sulfamethoxazole are represented by positive and negative values, respectively. *uckl1b* stands for uridine-cytidine kinase-like 1; *uck2b* stands for uridine-cytidine kinase 2-B; *rrm2* stands for ribonucleotide reductase regulatory subunit M2; *GSTP1* stands for glutathione S-transferase pi 1; *gsta3* stands forglutathione S-transferase 3; *gsto2* stands forglutathione S-transferase omega 1; *rrm2b* stands forribonucleoside-diphosphate reductase subunit M2 B-like; *gstt2b* stands for glutathione S-transferase theta 2B; *ugt* stands for UDP-glucuronosyltransferase; *ugt2a1* stands for UDP-glucuronosyltransferase 2C1-ike; and *ces3* stands for carboxylesterase 5A-like.

After GO and KEGG analyses, we chose a few significantly expressed drug metabolism-related genes that were crucial to the research subject. Primers for these genes were then used to validate the RNA-seq results. A list of all primer sequences can be found in Table 3. According to the data, the RNA-seq results were consistent with the up- or downregulation of the genes (Figure 4). These results showed that the RNA-seq and qRT-PCR data were generally reliable.

## 4. Discussion

Illumina sequencing is a precise and powerful molecular genetic assay that makes it the perfect instrument for examining changes in sulfamethoxazole activity at the transcriptome level. The method is widely used in animal transcriptome studies [24], which has been successfully applied in the transcriptome analysis of flounder [25], Anguilla japonica [26], and Chinese mitten crab at present [27].

Sulfonamides are widely used in the aquatic industry, which achieve bacterial inhibition by interfering with the metabolism of folic acid in pathogenic bacteria. But sulfamethoxazole contamination in water creates a great threat to aquatic creatures. Sulfamethoxazole can have an impact on aquatic algae and hinder their growth when it is present in water [28]. Furthermore, it has been demonstrated that zebrafish exposed to various sulfamethoxazole doses for 21 days suffered significant liver damage, including noticeable epithelial detachment, cytoplasmic hyalinization, vacuolization, and tissue abnormalities. According to transcriptomics, the drug’s effects also altered the liver’s ability to convert fat, leading to metabolic problems and alterations in relevant genes [29].

### 4.1. Glucose and Lipid Metabolism

Glucuronidation is an important detoxification pathway for organic pollutants in fish. In the present experiment, we found that after the action of sulfamethoxazole, some EDGs were assigned to energy metabolism-related pathways, including glucuronidation, oxidative phosphorylation, and succinate metabolism. Among them, UDP-glucuronosyltransferase (*ugt*), a member of the UDP-glycosyltransferase family, and UDP-glucuronosyltransferase 2C1-like (*ugt2a1*) showed a significant upregulation. *ugt* catalyzes the phase II biotransformation reaction, where a lipophilic substrate couples with glucuronide to increase the water solubility of the metabolite, thus facilitating excretion into the urine or bile [30], which is essential for the elimination and detoxification of drugs and exogenous and endogenous compounds [31]. *Ugt* also catalyzes the glucuronidation of endogenous steroid hormones such as androgens and estrogens [32]. By catalyzing the glucuronidation of BA substrates, it contributes to bile acid (BA) detoxification [33]. The glucuronide pathway is a branch of glucose metabolism and its product, UDP-glucuronide, is detoxifying, mainly in the liver and erythrocytes. After the organism is stimulated by a drug, the drug molecules are absorbed to undergo biotransformation in the presence of body enzymes. Therefore, this transformation is carried out in large quantities after the drug acts on the liver, which increases the demand for the *UGT* family of enzyme systems. Currently, it has been reported that injection of alloxanamine 1254 or lindane (gamma-HCH) into the peritoneal cavity of flounder leads to elevated flounder *UGT1B* mRNA in the liver to facilitate drug metabolism in the liver [34]. *Ugt2a1* is annotated in GO for carbohydrate binding and glucuronosyltransferase activity. This is the same phenomenon noticed in the results of the present experiment, which may indicate the promotion of glucuronidation in the fish liver after drug action.

Lipids are the main source of energy in fish, and their uptake of lipids influences fish body functions. We found that carboxylesterase 5A- like(*ces3*) is also upregulated in response to drugs. *Ces3* is a protein-coding gene that has been implicated in hydrolytic enzyme activity and carboxylester hydrolase activity in GO annotation. At the same time, *ces3* is a member of the large family of carboxylesterases, which are responsible for the hydrolysis or esterification of various exogenous substances as well as endogenous substrates with ester, thioester, or amide bonds, and are involved in the metabolism of fatty acyl and cholesterol esters [35]. The activation of this gene after drug action contributes to the metabolism of complex lipids.

### 4.2. Antioxidant

Drug metabolism in the body generally relies on *glutathione-S-transferase* (*GST*), carboxylesterases, and cytochrome P450 enzymes [36]. Drug metabolism activates the antioxidant system for organismal defense, but fish have a limited response to external stimuli, which can cause cell death when the fish itself cannot resist the effects. In the present study, we found that *glutathione S-transferase pi 1*(*GSTP1*), *glutathione S-transferase θ 2 B*(*gstt2b*), *glutathione S-transferase 3*(*gsta3*), and glutathione S-transferase omega 1(*gsto2*) were upregulated in response to the drug sulfamethoxazole, which affects normal antioxidant processes in fish. The main function of *GST*s is to catalyze the binding of electrophilic groups of certain endogenous or exogenous hazardous substances to the sulfhydryl groups of reduced glutathione to form more soluble and non-toxic derivatives. *GST*s are directly related to the metabolism of exogenous substances and are involved in phase II biotransformations; they play a central role in the detoxification of heterogeneous substances by binding to reduced glutathione and at the same time make it easily excreted or catabolized by phase III metabolic enzymes [37]. *GST*s often perform their detoxification and antioxidant functions to protect organisms from damage caused by adversity when they encounter it. These variable genes in GO and KEGG annotations are involved in the innate immune system, glutathione coupling, glutathione transferase activity, and thioltransferase activity [38]. These genes are involved in the innate immune system and can directly influence the synthesis of glutathione, thereby contributing to the antioxidant process. An experiment in which zebrafish were exposed to different concentrations of endosulfan showed that *GST* activity in the liver of zebrafish was significantly higher than that of the control group [39]. The activities of *GST* and catalase (*CAT*) in zebrafish larvae were observed after exposure to a 2,4-D triple sublethal concentration for 48 h. The results showed that *GST* activity significantly increased (*p* < 0.05) at the lowest concentration tested of 2.5 mg/L (147.52 ± 2.76 μmol/min/mg protein) in comparison with the control group (134.17 ± 7.76 μmol/min/mg protein). In turn, *CAT* activity decreased (*p* < 0.05) in larvae treated with an intermediate concentration of 5 mg/L (2.46 ± 0.30 μmol/min/mg protein), also compared to the control group (4.30 ± 1.06 μmol/min/mg protein). This indicated that drug treatment induced oxidative stress in the liver, while it stimulated the upregulation of *GST* and promoted drug metabolism. In addition, we have found that *GSTP1* is associated with xenobiotic metabolism, and considering this study, the upregulation of *GSTP1* may drive glucose xenobiotic metabolism, which enhances the glucose xenobiotic pathway to maintain energy homeostasis to ensure proper energy supply for metabolism and to replenish glucose or glycogen overconsumed by glycolysis under stress.

Similarly, we found in our results that most of the changed genes were associated with nucleotide metabolism, nucleotide recycling, and DNA synthesis, such as *dypd uck2b*, and *rrm2*, which were all shown to be upregulated at the 6 d mark. This suggests that in the process of drug metabolism, the cell also undergoes the same metabolism to maintain the normal functioning of the individual in response to the stimulus.

## 5. Conclusions

We used sulfamethoxazole to stimulate channel catfish and obtained their liver transcriptome at different times for analysis and comparison. We found that some genes related to glycolipid metabolism and antioxidants showed significant changes. Upon stimulation, channel catfish may promote glucuronidation by regulating gene changes, while enhancing fatty acid metabolism to maintain normal detoxification processes. The antioxidant program was suppressed by drug action, but the genes related to nucleoside synthesis and metabolism were also altered at the same time to maintain the normal functioning of the organism to a certain extent. In the comparison among the groups, the genes promoting glucuronidation in the UDP-glycosyltransferase family were upregulated, ces3 involved in fatty acyl and cholesterol esters were upregulated, and *GST*, which is associated with the in vivo metabolism of drugs, was upregulated. The results of this study elucidated the differential changes in the liver gene transcriptome of the channel catfish under the action of sulfamethoxazole, revealed the mechanism of action of sulfamethoxazole, and provided a new study on changes in the liver genome induced by the action of the drug.

## Figures and Tables

**Table 1:** Oligonucleotide primers used to validate DEGs by qRT-PCR.

Gene Symbol	Description	Primer Name	Nucleotide Sequence (5′-3′)	Expected Product (bp)
*uckl1b*	uridine-cytidine kinase-like 1	uckl1b-F uckl1b-R	GCCTGTGTAAGGTGCTGTGT GCTGCTGCTGCTGCTCTATG	173
*uck2b*	uridine-cytidine kinase 2-B	uck2b-F uck2b-R	CATCCTCAACGGCGGCTTCA TCAGTGCGGTCTGCTGGACT	100
*rrm2*	ribonucleotide reductase regulatory subunit M2	rrm2-F rrm2-R	TCCACCGACAACACCTTCCG GGCAGTGGTCACAGGAGCAT	105
*GSTP1*	glutathione S-transferase pi 1	GSTP1-F GSTP1-R	GCTCTGCGGATCATGCTGTC TGCGTCCCAAATGCCTCAAA	178
*gsta3*	glutathione S-transferase 3	gsta3-F gsta3-R	AGCCAAGGAACGCACTCTTC GAGCACCTCCTCCAGCATGA	127
*gsto2*	glutathione S-transferase omega 1	gsto2-F gsto2-R	CCGTGGTTTGAGAGGGTGGA TGACTGCCTTCACTGCTGGA	112
*rrm2b*	ribonucleoside-diphosphate reductase subunit M2 B-like	rrm2b-F rrm2b-R	ACCATGCGAACCGACTCCAA CGACTGTCCAGAACGATGCC	175
*gstt2b*	glutathione S-transferase theta 2B	gstt2b-F gstt2b-R	ACCTGTCCTGGCAGCATTCA ACGGCAGCATCCATCTTGTC	118
*ugt*	UDP-glucuronosyltransferase	ugt-F ugt-R	GCCGTGTTCTGGACCGAGTT GCAGGAGGAAGGCGATGACA	118
*ugt2a1*	UDP-glucuronosyltransferase 2C1-like	ugt2a1-F ugt2a1-R	TGGCGATACACAGGCGAGAA GTCAGGCTGATCCGCAAACA	195
*ces3*	carboxylesterase 5A-like	ces3-F ces3-R	TGTTCGCACAGGCTCACCAA GCTTCTGAGGCAGCTCCACA	155

**Table 2:** Summary of statistical tables of base information for control (CK) and treated (T) groups.

Sample	Raw Data (bp)	Clean Data (bp)	Q20 (%)	Q30 (%)	N (%)	GC (%)
CK-1	8,528,218,500	8,455,531,252	8,312,672,702 (98.31%)	8,034,203,258 (95.02%)	88,081 (0.00%)	3,943,852,525 (46.64%)
CK-2	8,098,164,900	8,022,273,318	7,873,934,629 (98.15%)	7,590,887,887 (94.62%)	85,023 (0.00%)	3,885,940,274 (48.44%)
CK-3	7,866,796,200	7,790,326,860	7,647,431,709 (98.17%)	7,375,843,021 (94.68%)	80,343 (0.00%)	3,689,368,216 (47.36%)
T1-1	8,295,867,300	8,217,440,673	8,051,283,794 (97.98%)	7,743,396,884 (94.23%)	86,100 (0.00%)	3,870,550,118 (47.10%)
T1-2	7,531,641,600	7,457,377,413	7,295,029,054 (97.82%)	6,998,206,939 (93.84%)	77,000 (0.00%)	3,534,572,880 (47.40%)
T1-3	7,386,847,200	7,322,938,214	7,188,711,492 (98.17%)	6,930,035,630 (94.63%)	76,341 (0.00%)	3,486,858,742 (47.62%)
T2-1	7,918,878,000	7,834,621,505	7,681,087,705 (98.04%)	7,395,471,402 (94.39%)	80,981 (0.00%)	3,668,850,749 (46.83%)
T2-2	7,269,383,100	7,204,736,382	7,067,332,104 (98.09%)	6,806,621,229 (94.47%)	73,881 (0.00%)	3,398,786,165 (47.17%)
T2-3	8,150,140,800	8,070,530,230	7,925,943,852 (98.21%)	7,650,521,989 (94.80%)	84,886 (0.00%)	3,763,940,093 (46.64%)
T3-1	6,986,844,300	6,926,075,339	6,798,229,961 (98.15%)	6,544,811,236 (94.50%)	71,170 (0.00%)	3,232,926,751 (46.68%)
T3-2	7,957,478,400	7,900,931,726	7,767,533,892 (98.31%)	7,497,502,331 (94.89%)	82,047 (0.00%)	3,703,797,826 (46.88%)
T3-3	7,329,309,900	7,265,578,461	7,146,030,274 (98.35%)	6,900,631,072 (94.98%)	75,379 (0.00%)	3,418,766,468 (47.05%)

**Table 3:** Statistical results of trimmed read mapping with a reference genome.

Sample	Total	Unmapped (%)	Unique_Mapped (%)	Multiple_Mapped (%)	Total_Mapped (%)
CK-1	56,593,710	4,177,297 (7.38%)	49,626,790 (87.69%)	2,789,623 (4.93%)	52,416,413 (92.62%)
CK-2	53,663,248	4,566,882 (8.51%)	46,317,822 (86.31%)	2,778,544 (5.18%)	49,096,366 (91.49%)
CK-3	52,108,754	4,102,178 (7.87%)	45,449,379 (87.22%)	2,557,197 (4.91%)	48,006,576 (92.13%)
T1-1	54,955,458	4,414,562 (8.03%)	48,089,747 (87.51%)	2,451,149 (4.46%)	50,540,896 (91.97%)
T1-2	49,875,330	4,236,696 (8.49%)	43,303,053 (86.82%)	2,335,581 (4.68%)	45,638,634 (91.51%)
T1-3	48,971,260	3,965,702 (8.10%)	42,764,114 (87.32%)	2,241,444 (4.58%)	45,005,558 (91.90%)
T2-1	52,463,010	4,222,162 (8.05%)	45,801,883 (87.30%)	2,438,965 (4.65%)	48,240,848 (91.95%)
T2-2	48,161,994	4,038,301 (8.38%)	42,007,026 (87.22%)	2,116,667 (4.39%)	44,123,693 (91.62%)
T2-3	54,071,356	4,310,287 (7.97%)	47,335,777 (87.54%)	2,425,292 (4.49%)	49,761,069 (92.03%)
T3-1	46,375,682	3,417,071 (7.37%)	40,595,071 (87.54%)	2,363,540 (5.10%)	42,958,611 (92.63%)
T3-2	52,855,970	3,919,932 (7.42%)	46,517,939 (88.01%)	2,4180,99 (4.57%)	48,936,038 (92.58%)
T3-3	48,693,526	3,443,780 (7.07%)	42,888,480 (88.08%)	2,361,266 (4.85%)	45,249,746 (92.93%)

## Data Availability

Data generated or analyzed during this study are provided in full within the published article.
